# Synthesis and field emission studies of tower-like GaN nanowires

**DOI:** 10.1186/1556-276X-9-607

**Published:** 2014-11-08

**Authors:** Yihe Liu, Xianquan Meng, Xiang Wan, Zelong Wang, Huihui Huang, Hao Long, Zengcai Song, Guojia Fang

**Affiliations:** 1 Key Laboratory of Artificial Micro-and Nanostructures Ministry of Education and School of Physics and Technology, Wuhan University, Luojia Hill, Wuhan 430072, Hubei, People's Republic of China

**Keywords:** Tower-like nanowires, Chemical vapor deposition, TEM, Field emission

## Abstract

Tower-like GaN nanowires were successfully fabricated on Au-coated Si substrates by chemical vapor deposition. The tower-like nanowire consisted of a nanowire at the center and microcrystal layers stacked one by one around the nanowire. The tower-like nanowires grew along the [0001] direction, and the exposed surfaces of the microcrystal layers are $\left(10\bar{1}1\right)$ and $\left(10\bar{1}\bar{1}\right)$ facets. The growth mechanism of the tower-like GaN nanowires was proposed. The field emission property of tower-like GaN nanowires was tested. Due to the sharp tips, nearly vertical alignment and rough surfaces caused by the microcrystal layers, the tower-like GaN nanowires show excellent performance in field emission with a turn-on field of 2.44 V/μm which is lower than those of other GaN one-dimensional (1D) nanomaterials.

**PACS:**

81.15.Gh; 68.37.Lp; 68.37.Vj

## Background

One-dimensional (1D) nanomaterials are excellent candidates for nanophotonics, nanoelectronics, and biosensing devices [1-3]. Compared to 2D films, such 1D nanomaterials provide the direct conduction paths for carriers to transport. Several studies have revealed that they possess much larger diffusion length of minority carrier [4] and high transportation rate [5-7]. With wide and direct bandgap, large carrier mobility, high thermal, and chemical stability as well as capability of n- or p-type doping [8,9], GaN has attracted great research interests and found the applications in high-performance optoelectronics devices [10]. GaN is also believed to be an excellent field electron emitter as it owns a low electron affinity of 2.7 to 3.3 eV which would cause the formation of a lower emission potential barrier.

During the past few years, some GaN 1D nanomaterials have been fabricated, such as stacked-cone [11-13] and zigzag GaN nanowires [14], GaN nanorods [15], nanobelts [16], nanosheets [17], and tower-like GaN nanostructures [18]. Although the catalyst-assisted vapor-liquid-solid (VLS) growth mechanism is most widely exploited to explain the fabrication of 1D nanomaterials such as nanowires or nanorods, the growth mechanism of GaN 1D nanomaterials with other morphologies is still unclear. It is important to study the formation mechanism of these nanostructures for developing the new nanoscale functional materials.

Here, we report the successful fabrication of tower-like GaN nanowires by chemical vapor deposition. The tower-like GaN nanowires show excellent performance in field emission test, making them a competitive candidate for preparing field electron emission devices.

## Methods

The tower-like GaN nanowires were synthesized by chemical vapor deposition. Au film of 6 to 10 nm in thickness was deposited on the Si substrate by RF magnetron sputtering. The Au-coated Si substrate was located about 1 cm downstream to the 99.99% Ga source in an alumina boat, and both are placed in a tube within a furnace. The tube was pumped and gradually heated to 850°C at a rate of 25°C/min. Then, NH_3_ gas was introduced at a flow rate of 120 sccm, and the pressure in the tube was 52 Pa during the growth process. The growth temperature was maintained at 850°C for a desired growth time. Finally, the system was cooled down to room temperature in vacuum. GaN products were obtained on the Si substrate.

The products were characterized by scanning electron microscopy (SEM; JSM-6700), field-emission transmission electron microscopy (TEM; JEM 2010 FEF), and field emission (FE).

## Results and discussion

**Figure 1:**
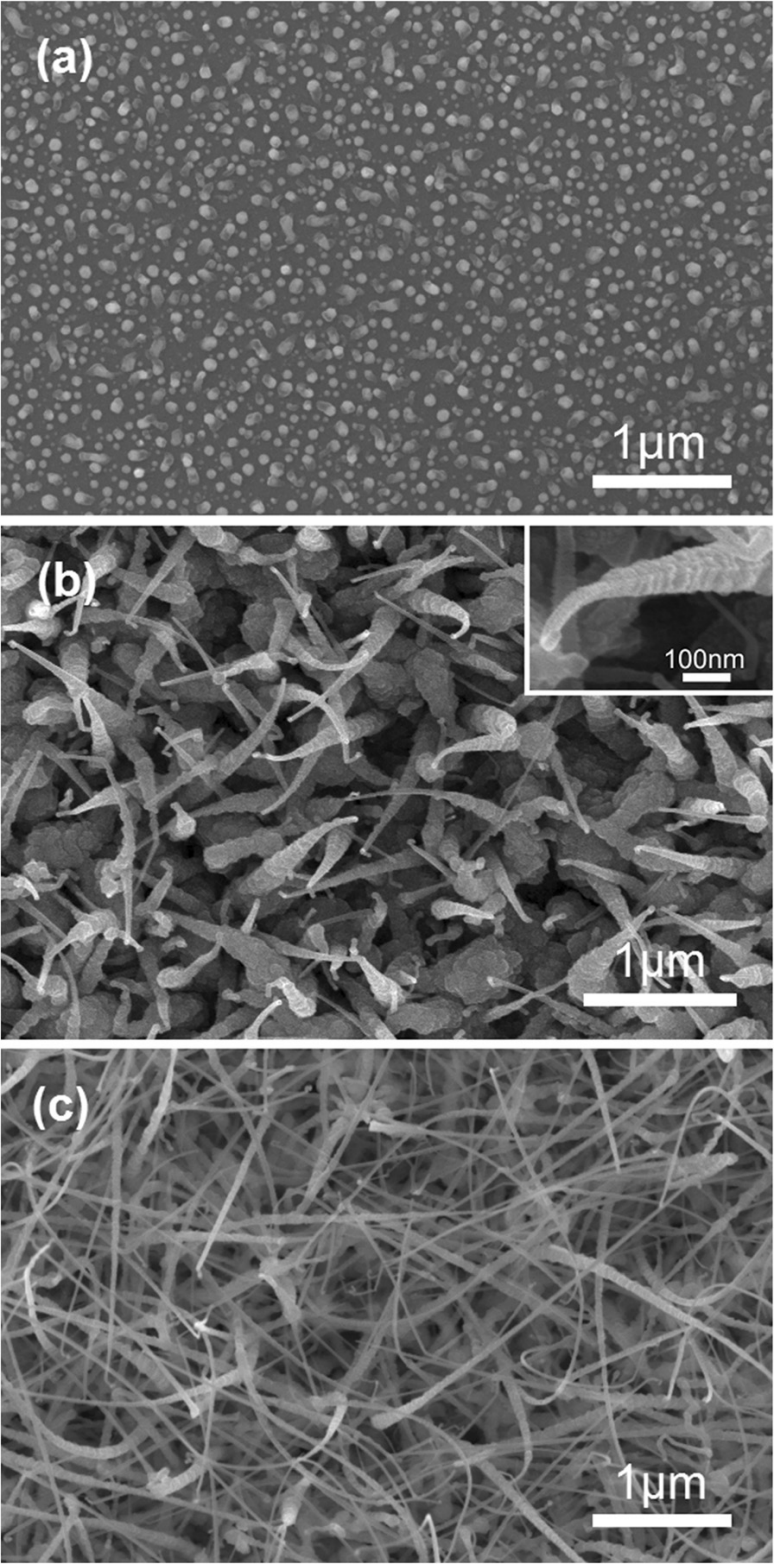
**SEM images of the GaN products with various growth time. (a)** 3 min, **(b)** 10 min, and **(c)** 40 min, respectively. Inset in Figure 1b shows the magnified SEM image of a typical tower-like GaN nanowire grown for 10 min.

Figure 1a shows the SEM image of the GaN products grown for 3 min. It reveals the initial growth state of tower-like GaN nanowires. In the stage of heating, the sputtered Au film transforms into small Au islands, which range from 10 to 80 nm in size. The majority of the tower-like GaN nanowires grow out from Au islands of 40 to 60 nm in size. The density of the Au islands is about 8.5 × 10^9^/cm^2^.

Tower-shaped products which have grown for 10 min are shown in Figure 1b. They are about 1 μm in length with decreasing diameters along the growth direction. The bottoms of the tower-like GaN nanowires are about 150 to 200 nm in width, while the top tips are 40 to 60 nm. The magnified SEM image of a single typical tower-like nanowire grown for 10 min is displayed in the inset of Figure 1b. It indicates that the tower-like GaN nanowires consist of two types of structures with distinct morphologies. The first one is the wire-like structure at the center, and the second is the microcrystal layers around the nanowire with the base microcrystal layer having a diameter larger than those of stacked above. Au nanoparticles can be clearly seen at the tip of the tower-like nanowires, suggesting that the axial growth of the tower-like nanowires is based on the VLS mechanism [19]. The radial growth of the tower-like GaN nanowires is based on vapor-solid (VS) mechanism.

The tower-like GaN nanowires in Figure 1b are nearly vertical to the substrate. Similar vertically standing GaN nanowires grown with Au catalyst have also been fabricated under low pressure by Hou et al. [20]. As the reactor was kept at reduced pressure (approximately 52 Pa), radicals of Ga and N were expected to be partly bumped away and their quantity would be reduced significantly. The growth rate of the nanowires was then reduced greatly which would promote vertical alignment of the nanowires. Also, the microcrystal layers around the central wire were helpful for the standing of the wires. Under low pressure, Ga droplet which transported to the substrate was expected to be converted into GaN nanodots. The GaN nanodots acted as seed (or) nucleation for subsequent growth of GaN nanowires via VS mechanism with slow growth rate, which promoted vertical standing of the nanowires [21].Figure 1c displays the GaN products grown for 40 min. It shows that the products have the tail-tapered morphology and their length is increased. In addition, some long nanowires can be observed. As growth time increases, more nanowires may grow out from Au islands surviving from the early growth. Furthermore, Ga vapor is transported to Si substrate continuously. A portion of Ga accumulates at Si substrate and forms islands which act as seed nucleation for the nanowire growth. These lead to an increase in density of the nanowires. In addition, non-vertical standing nanowires and the longer length of nanowires make their density look like to be increased. With growth duration increasing to 40 min at a rather high temperature (approximately 850°C), Au nanoparticles at the tips disappear due to volatilization or desorption and then, it is hard to see nanoparticles at the tips of the nanowires in Figure 1c.

**Figure 2:**
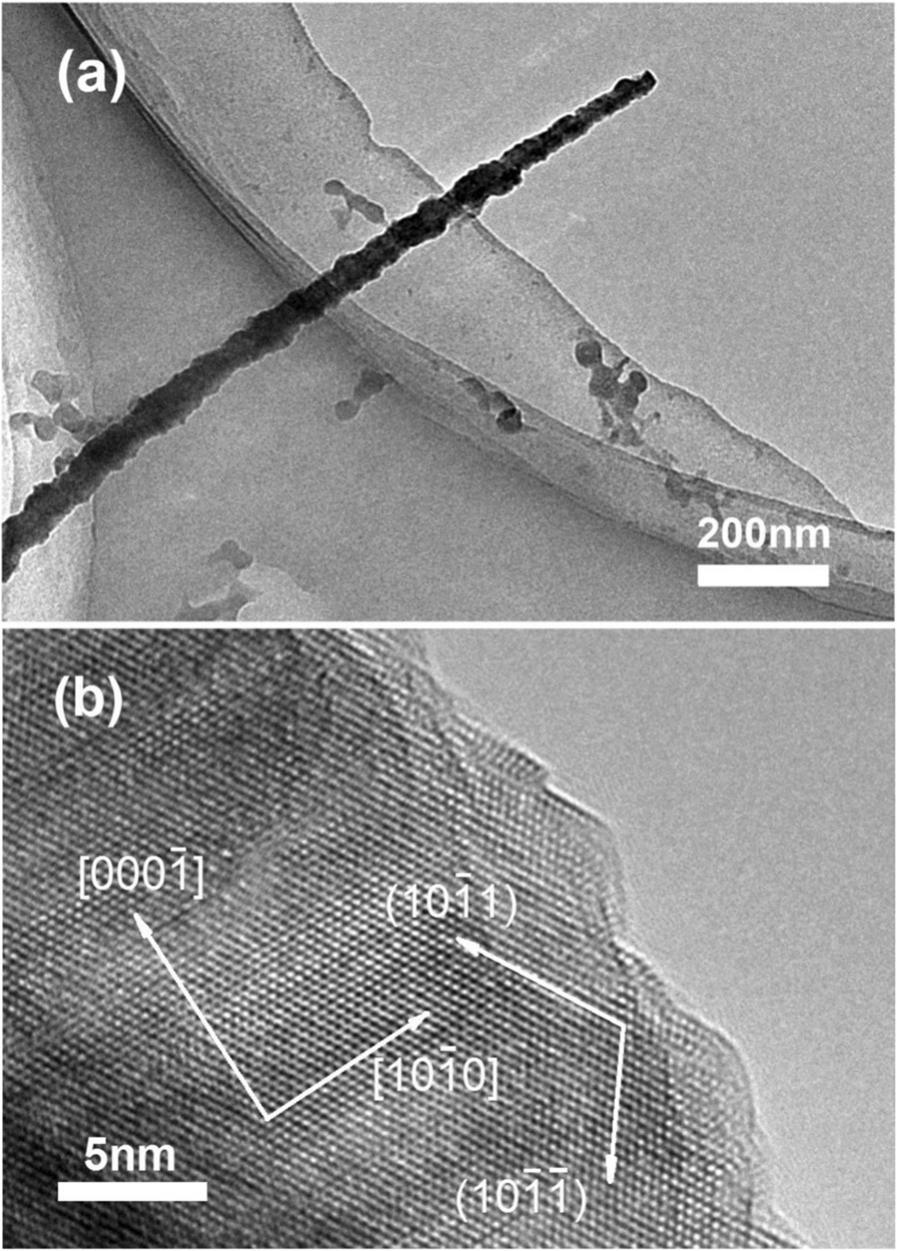
**The TEM image and HRTEM image of a single truncated tower-like GaN nanowire. (a)** The TEM image of the nanowire grown for 40 min. **(b)** HRTEM image of the nanowire grown for 40 min.

Figure 2a shows a TEM image of a single truncated GaN nanowire grown for 40 min with microcrystal layers on sidewalls. The nanowire shows tail-tapered morphology because the top and wide base of the wire were broken up in the process for preparing high-resolution transmission electron microscopy (HRTEM) sample. And Figure 2b is the HRTEM image of the nanowire grown for 40 min. The clear lattice fringes confirm that the tower-like nanowire is single crystal. As displayed by the picture, the GaN nanowire grows along the [0001] direction. The microcrystal layers around the wire are exposed by $\left(10\bar{1}1\right)$ and $\left(10\bar{1}\bar{1}\right)$ facets.

**Figure 3:**
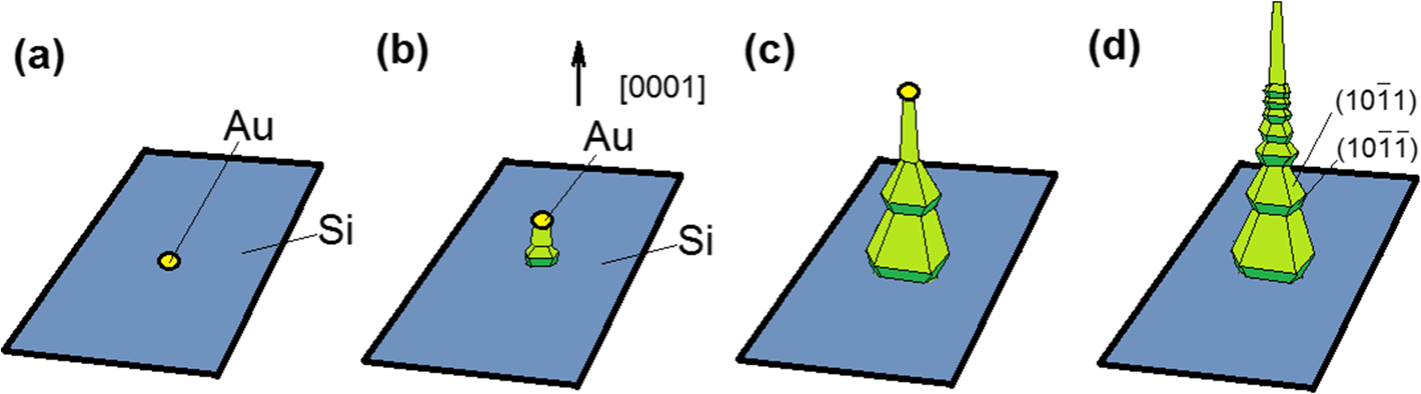
**Formation schematic diagram of the tower-like GaN nanowires. (a)** Au film transforms into Au islands. **(b)** The nanowire grows out. **(c)** The tower-like GaN nanowire forms. **(d)** The GaN nanowire grows longer with features of tail-tapered and tower-like in base part.

Now we suggest a plausible growth mechanism to explain the formation of tower-like GaN nanowires. As displayed in Figure 3a, the Au film transforms into nanoscale islands or particles. The islands acted as catalyst decomposing and dissolved the gas phase reaction species. When the gas phase species is supplied continuously, the super saturation at the liquid-solid interface led to the axial growth of nanowires. The gas phase reaction species may arrive at: (1) the nanowire tip through adsorption on the particle, (2) the nanowire sidewalls through direct adsorption [22], or (3) the nanowire base via substrate surface diffusion after the first adsorption on the growth substrate [23]. Ga and N radicals followed two pathways for the incorporation onto the growth site: the first way is the adatom diffusion through catalyst droplet, and the second is the adatom migration on the surface of the droplet and sidewalls of the wire through the interface [22]. As the Ga and NH_3_ supply continued, the supersaturation at the liquid-solid interface led to the axial growth of nanowires along the preferred [0001] direction, as shown in Figure 3b.The gas species arriving at the nanowire bases and sidewalls as mentioned in the above cases (2) and (3) would diffuse further along the nanowire surface. If they diffused to the growth site under catalyst droplet, axial growth of nanowires was promoted. When the nanowires grew long enough, especially the length of nanowires is larger than the diffusion length, most of Ga and N radicals could not reach the growth site, and they would stay and crystallize on the side faces of the nanowires and form the microcrystal layers. The tower-like GaN nanowires began to be formed as displayed in Figure 3c.

It has been known that the diffusivity of adatoms on different surfaces of GaN nanowires can be quite different and the presence of adsorbates will further change the characteristics of the surfaces. However, under a rather low Ga source condition, adatom diffusion on faces $\left(10\bar{1}1\right)$ was limited and most of the adatoms could not reach the catalyst at the tip, so the surfaces became rough, leading to coarse and corrugated side surfaces. Further roughening of the side faces led to the development of $\left(10\bar{1}1\right)$ and $\left(10\bar{1}\bar{1}\right)$ facets and then close packing, as shown in Figure 2b. Surface reconstruction by the dimerization of atoms readily occurred on these surfaces and would further reduce the energy of these surfaces. We note that $\left(10\bar{1}1\right)$ planes are Ga polar, while $\left(10\bar{1}\bar{1}\right)$ planes are N terminated, so the $\left(10\bar{1}1\right)$ planes have lower formation energies and higher adatom diffusivity and they are more likely to develop at the expense of the $\left(10\bar{1}\bar{1}\right)$ planes [13] shown in Figure 3d.

As the growth of GaN nanowires went on, more nanowires grew out and the density of nanowires increased. The gas phase reaction species, which were adsorbed on the substrate and diffused to the nanowire base, decreased gradually and vanished finally. Hence, the contribution to the formation of microcrystal layers diminished. So the feature of tower-like nanowires began to disappear. At the same time, gas phase reaction species arriving at the nanowire tip and sidewalls increased, so their contribution to nanowire growth became more important. If the distance between the adsorbed species on sidewalls and the growth site is longer than the diffusion length of Ga atoms, nanowire growth showed the tapering effect. [21] In this case, the radicals of Ga and N stayed on the side faces only caused the tapering effect and could not form microcrystal layers, so the products grown with longer duration contained both features of tail-tapered and tower-like in base part, which is shown in Figure 3d. Because the top and base of the product were broken up, only the image of the upper part of the product is shown in Figure 2a.

**Figure 4:**
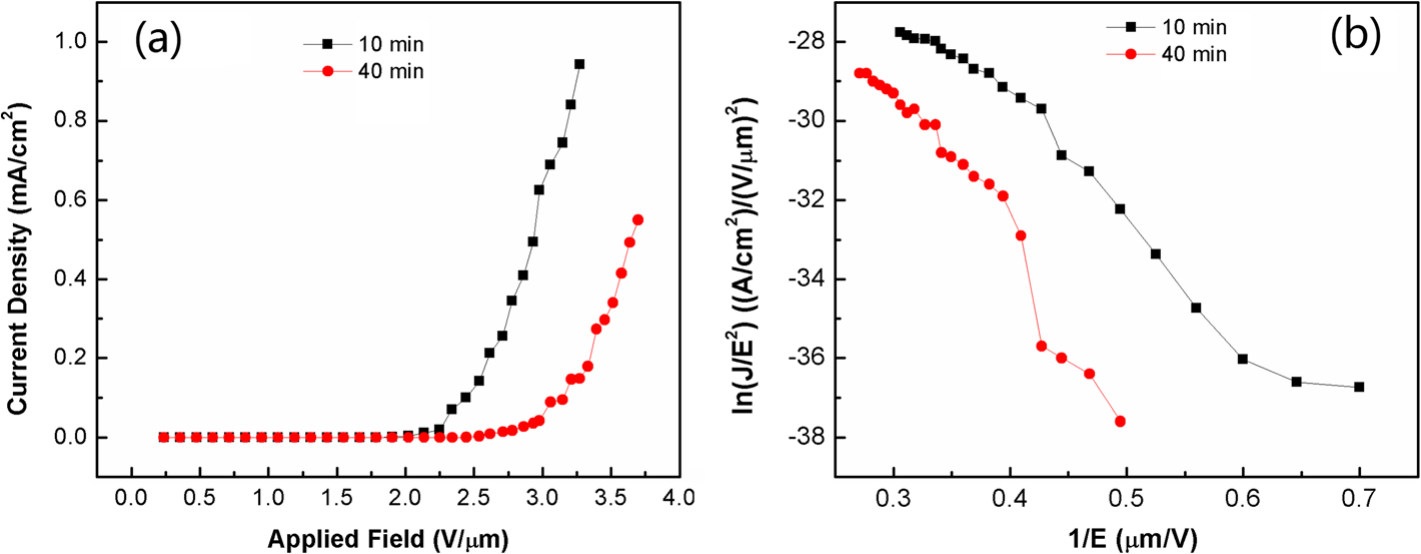
**The applied field dependence of the emission current density J and the corresponding Fowler-Nordheim plot. (a)***J*-*E* curves of the tower-like GaN nanowires grown for 10 and 40 min. **(b)** The corresponding Fowler-Nordheim plot.

Figure 4a shows the applied field dependence of the emission current density *J* of the GaN nanowires grown for 10 and 40 min, respectively. The applied field was sweeping from 0.24 to 3.70 Vμm^-1^ (applied voltage from 50 to 771 V) with anode-sample distance of 210 μm. The test was performed in a vacuum chamber under a pressure of 2 Pa at room temperature.

Here, we define the turn-on field (*E*_to_) as the electric fields required to produce a current density of 0.1 mAcm^-2^. The *E*_to_ of the tower-like GaN nanowires grown for 10 min is about 2.44 Vμm^-1^, which is much lower than the previous reported *E*_to_ of 21 Vμm^-1^[24] and 4.3 Vμm^-1^[25] for GaN nanowires under similar test environment. Also, Kimura et al. performed field emission works on bulk GaN or GaN nanorods with *E*_to_ of 4.6 Vμm^-1^[26], 12.4 Vμm^-1^[27], and 5 Vμm^-1^[28], respectively. The emission current density of the tower-like nanowires grown for 10 min increased rapidly to 1 mA/cm^2^ under an applied field of 3.3 Vμm^-1^. The *E*_to_ of the GaN nanowires grown for 40 min is about 3.2 Vμm^-1^ shown in Figure 4a.

Fowler-Nordheim (FN) theory was used to analyze the emission characteristics. According to the FN electron emission theory, emission current density can be expressed as:

$$J=A\left(E^{2}\beta^{2}/\Phi \right)\;\exp\left(-B\Phi^{3/2}/E\beta \right)$$

where *J* is the emission-current density, *A* = 4.43 × 10^-22^ AV^-2^ eV, *B* = 6.83 × 10^9^ VeV^-3/2^ m^-1^, *E* is the local applied field (where *E* = *V*/*d*, and *d* is the anode-sample distance), *Φ* is the work function (*Φ*_GaN_ = 4.1 eV). *β* is a field enhancement factor dependent on emitter geometry. Figure 4b shows the corresponding FN plots obtained at *d* = 210 μm for the tower-like GaN nanowires. By plotting ln (*J*/*E*^2^) versus *E*^-1^, a straight line was obtained. The linearity of the two curves implies that the FE from these tower-like nanowires follows FN theory. The deviations in the measurement in the low field regime are due to the detection limit (about 1 pA) of our measuring system.

From the slope of the FN plots, the field enhancement factor *β* of tower-like GaN nanowires grown for 10 min is estimated to be approximately 2,487, and that of the GaN nanowires grown for 40 min is about 1,601. In comparison with previous works on other GaN nanomaterials, our tower-like GaN nanowires show a relatively larger *β* value. Generally, the large *β* value is corresponding to the low turn-on field and large emission current density in field emission tests.

It is known that the field enhancement factor *β* is related to emitter geometry, crystal structure, and the spatial distribution of emitting centers. It can be expressed as *β* = *h*/*r*, where *h* is the height and *r* is the radius of curvature of an emitting center. Thus, the emission current of materials with elongated geometry and sharp tips can greatly be increased [29]. An insufficiently smooth surface morphology such as being roughened locally by surface damages may also enlarge *β*[30-32]. Both of sharp tips and surface roughness of our tower-like nanowires provide sufficiently high geometry enhancement. Li et al. pointed out that the better alignment of emitters, the larger the emitting current density [33]. Hence, the sharp tips of nanowires, surface roughness of the microcrystal layers, and nearly vertical alignment contribute to the excellent FE performance of tower-like nanowires.

For the GaN nanowires grown for 40 min, their *β* value is smaller than that of tower-like nanowires because of their non-vertical standing and relatively smooth surface, although they are longer and sharper than the tower-like nanowires.

## Conclusions

Tower-like GaN nanowires were successfully fabricated on Si substrate by chemical vapor deposition. The tower-like GaN nanowires show hexagonal structure and grow along [0001] direction with high crystalline quality. The tower-like GaN nanowire consists of a nanowire at the center and microcrystal layers stacked one by one around the wire with decreasing diameter along the growth direction. The growth of the GaN nanowire is based on VLS mechanism. The microcrystal layers are exposed with $\left(10\bar{1}1\right)$ and $\left(10\bar{1}\bar{1}\right)$ facets, grown based on VS mechanism. Tower-like GaN nanowires behaved very well in field emission test which owned a low turn-on field of 2.44 V/μm and generated the current density of 1 mA/cm^2^ under an applying field of 3.3 V/μm. The sharp top tips of tower-like nanowires, surface roughness of microcrystal layers, and the nearly vertical alignment all contribute to the excellent FE performance.

## Abbreviations

1D: one-dimensional; SEM: scanning electron microscopy; TEM: transmission electron microscopy; VLS: vapor-liquid-solid; VS: vapor-solid; FE: field emission.

## Competing interests

The authors declare that they have no competing interests.

## Authors' contributions

LYH performed the experiments, analyzed the data, and drafted the manuscript. The whole project was under the direction of MXQ, who designed the experiments and revised the manuscript. WX and WZL participated in the synthesis and characterization of the samples. HH, LH, SZC, and FGJ participated in the preparation of Au catalyst film and FE tests. All authors read and approved the final manuscript.
